# Supplementary material for: Complex Formation of Ag+ and Li+ with Host Molecules Modeled on Intercalation of Graphite
Source: Molecules. 2024 Aug 23;29(17):3987. doi: 10.3390/molecules29173987 (PMC11396304; doi:10.3390/molecules29173987)
Supplement: Supplementary file 1 [file molecules-29-03987-s001.zip › molecules-3163790-supplementary.pdf]

Supporting Information

Complex formation of Ag^+ and Li^+ with host molecules modeled on intercalation of graphite

Yuriko Uetake and Hiroyuki Takemura *
Department of Chemical and Biological Sciences,
Faculty of Science, Japan Women's University,
Mejirodai 2-8-1, Bunkyo-ku, Tokyo 112-8681, Japan;
yuriko.uetake@gmail.com
* Correspondence: takemurah@fc.jwu.ac.jp; Tel.: +81-3-5981-3664

1. NMR spectra of compound **2**.

Figure S1 ^{1}H-NMR spectrum of compound **2** ($CDCl_3$).

Figure S2 ^{13}C-NMR spectrum of compound **2** ($CDCl_3$).

2. Mass spectra of compound **2**.

Figure S3 HR-FAB MS spectrum of compound **2**.

Figure S4 Expanded HR-FAB MS spectrum of compound **2**.

3. NMR titrations

Figure S5 (a) ^{1}H NMR spectra of pyrene and pyrene-$AgClO_4$ mixtures (expanded)
Solvent: THF-d_8.
(b) Titration curve.

Figure S6 ^{1}H NMR spectra of compound **1** and **1**-$AgClO_4$ mixture (expanded).
Solvent: THF-d_8

Figure S7 (a) Titration curve of compound **1** and $AgClO_4$ fitted using the nonlinear square method.
(b) Job plots.

Figure S8 (a) ^{1}H NMR spectra of pyrene and pyrene-$LiClO_4$ mixture (expanded). Solvent: THF-d_8.

(b) Titration curve.

Figure S9 (a) ^{1}H NMR spectra of compound **1** and **1**-$LiClO_4$ mixture (expanded). Solvent: THF-d_8.

(b) Titration curve.

Figure S10 (a) ^{1}H NMR spectrum of compound **2** and **2**-$AgClO_4$ mixture (expanded). Solvent: THF-d_8

(b) Titration curve.

Figure S11 (a) ^{1}H NMR spectra of compound **2** and **2**-$LiClO_4$ mixture (expanded). Solvent: THF-d_8.

(b) Titration curve.

4. DFT Calculations. Cartesian coordinates of **1** and **2**.

1. NMR spectra of compound 2.

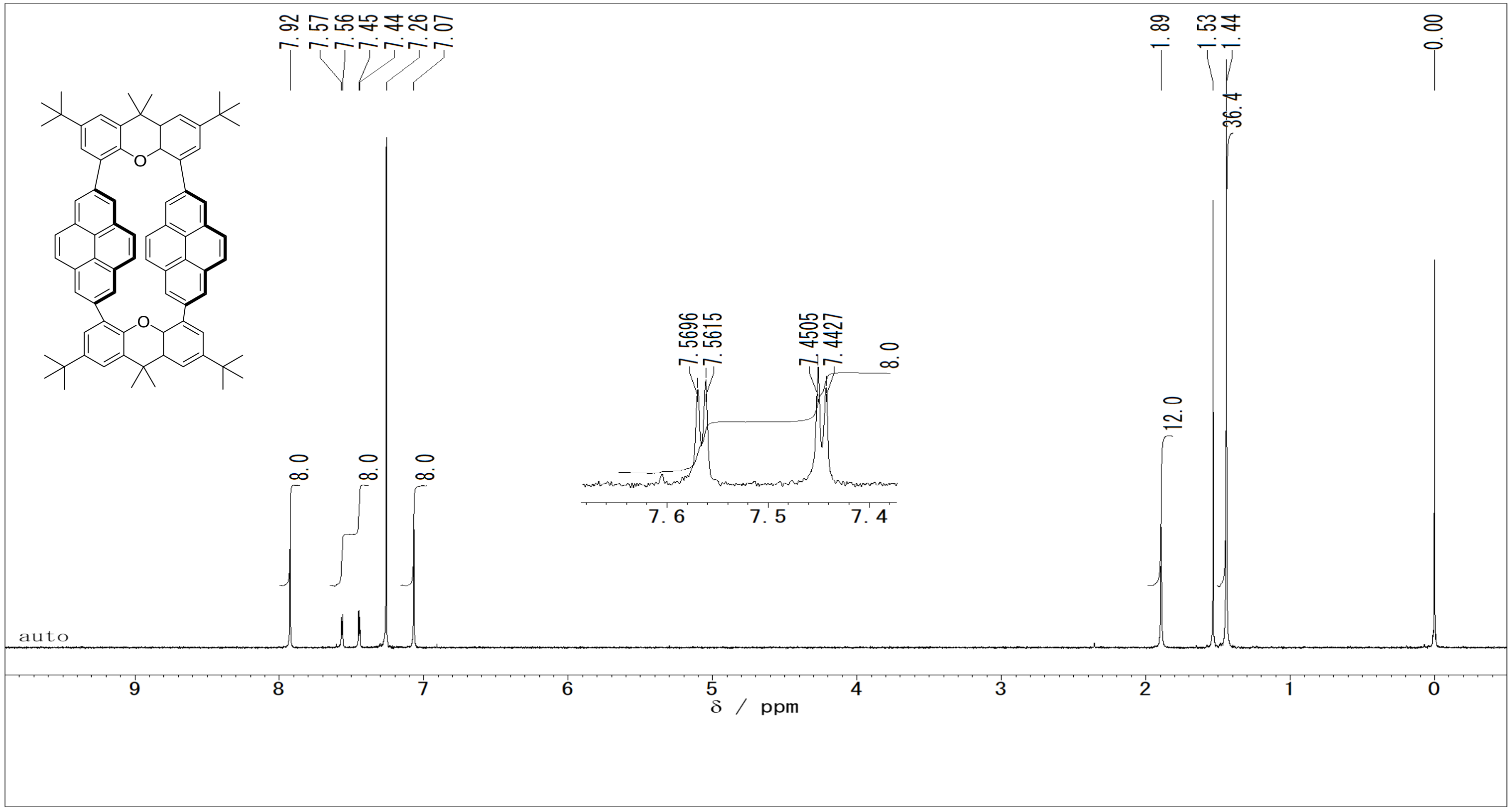

Figure S1 ^{1}H-NMR spectrum of compound **2** ($CDCl_3$).

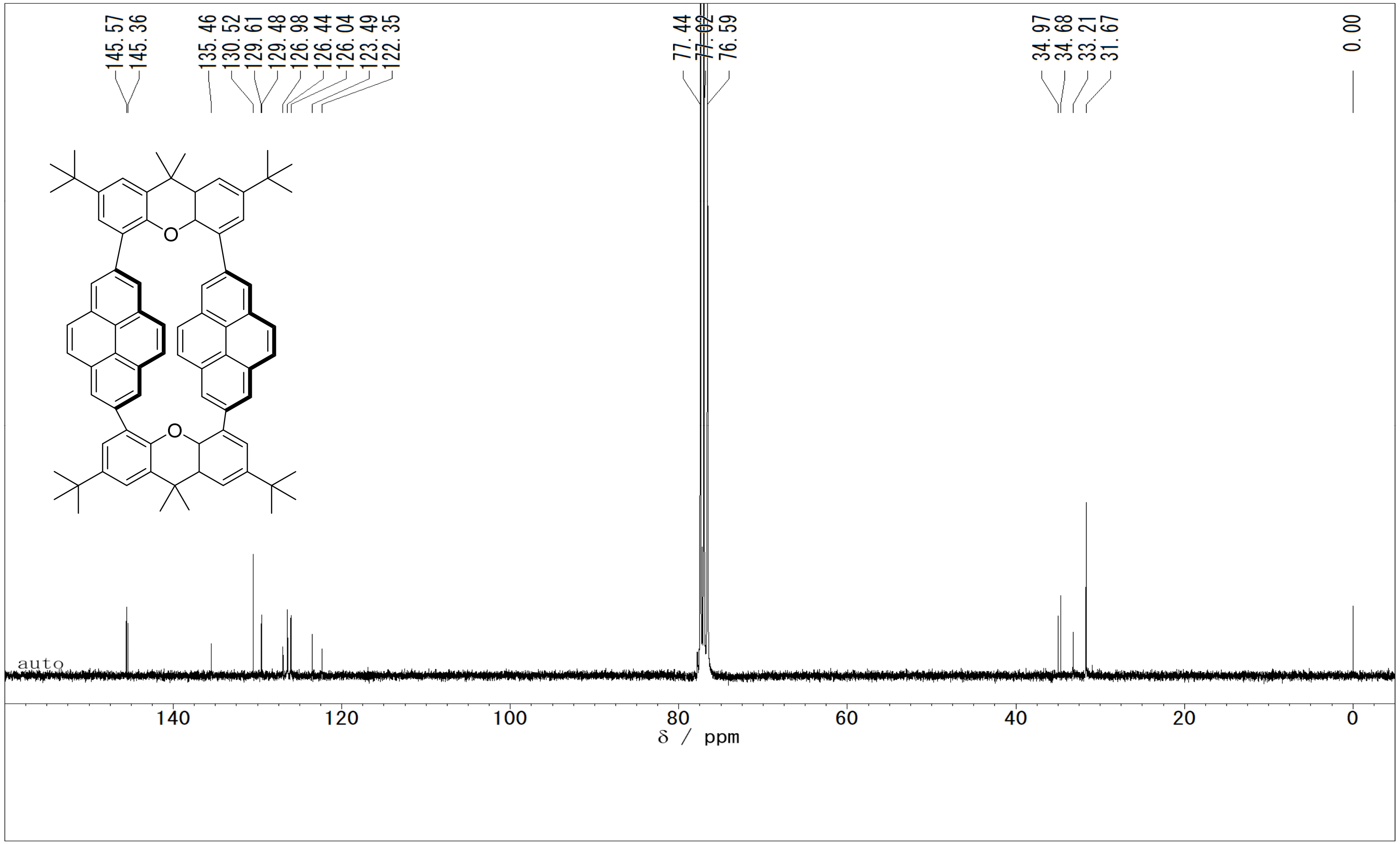

Figure S2 ^{13}C-NMR spectrum of compound **2** ($CDCl_3$).

2. Mass spectra of compound 2.

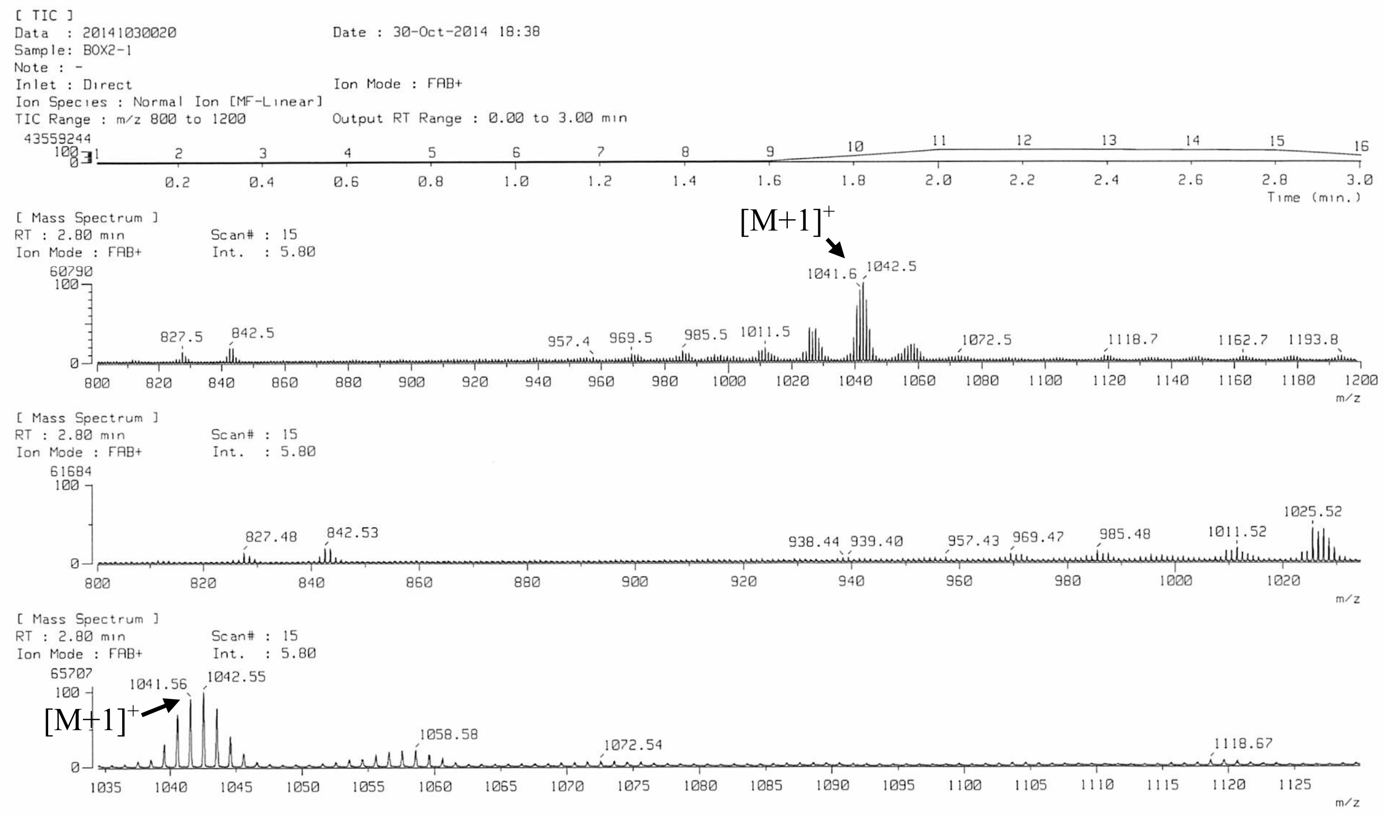

Figure S3 HR-FAB MS spectrum of compound **2**.

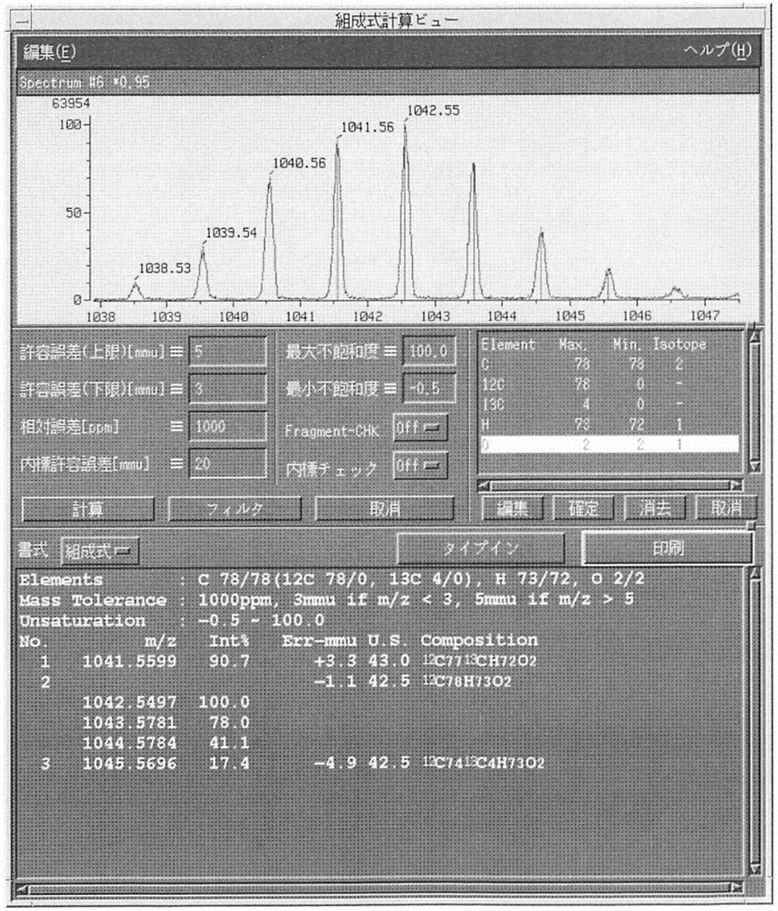

Figure S4 Expanded HR-FAB MS spectrum of compound **2**.

3. NMR titrations

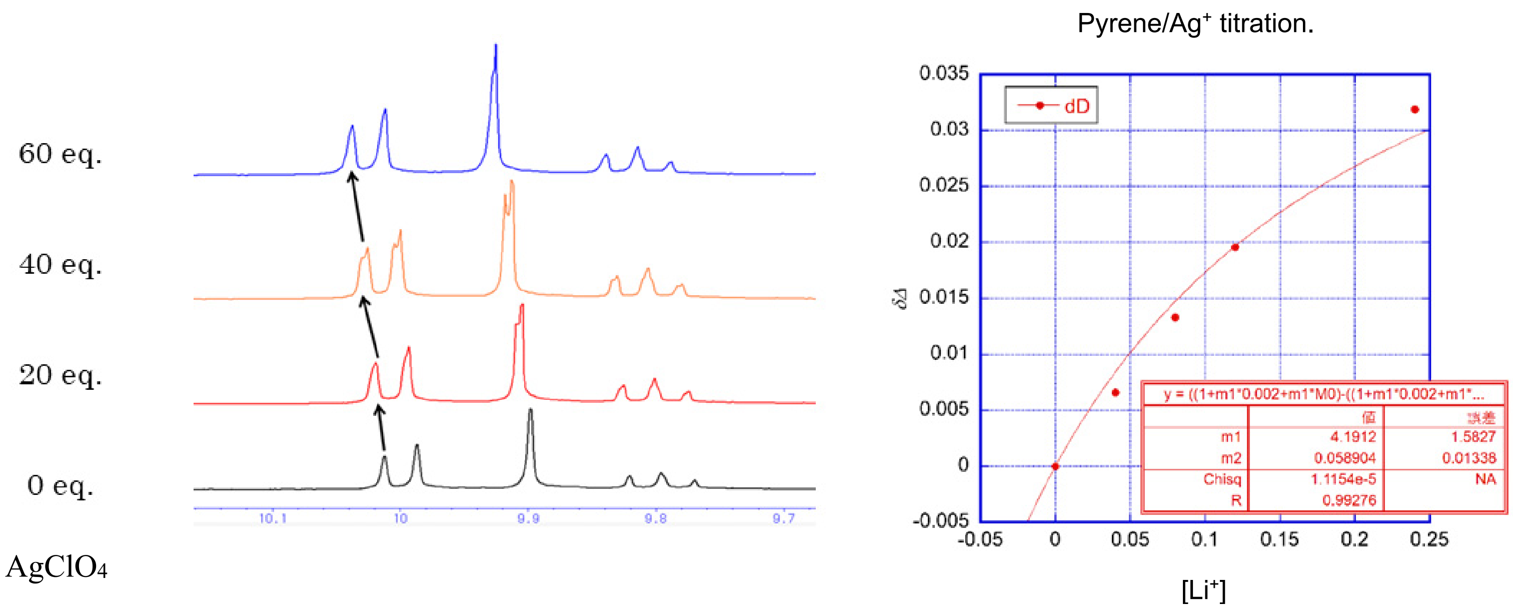

(a) (b)

Figure S5 (a) ^{1}H NMR spectra of pyrene and pyrene-$AgClO_4$ mixture (expanded). Solvent: THF-d_8.

(b) Titration curve.

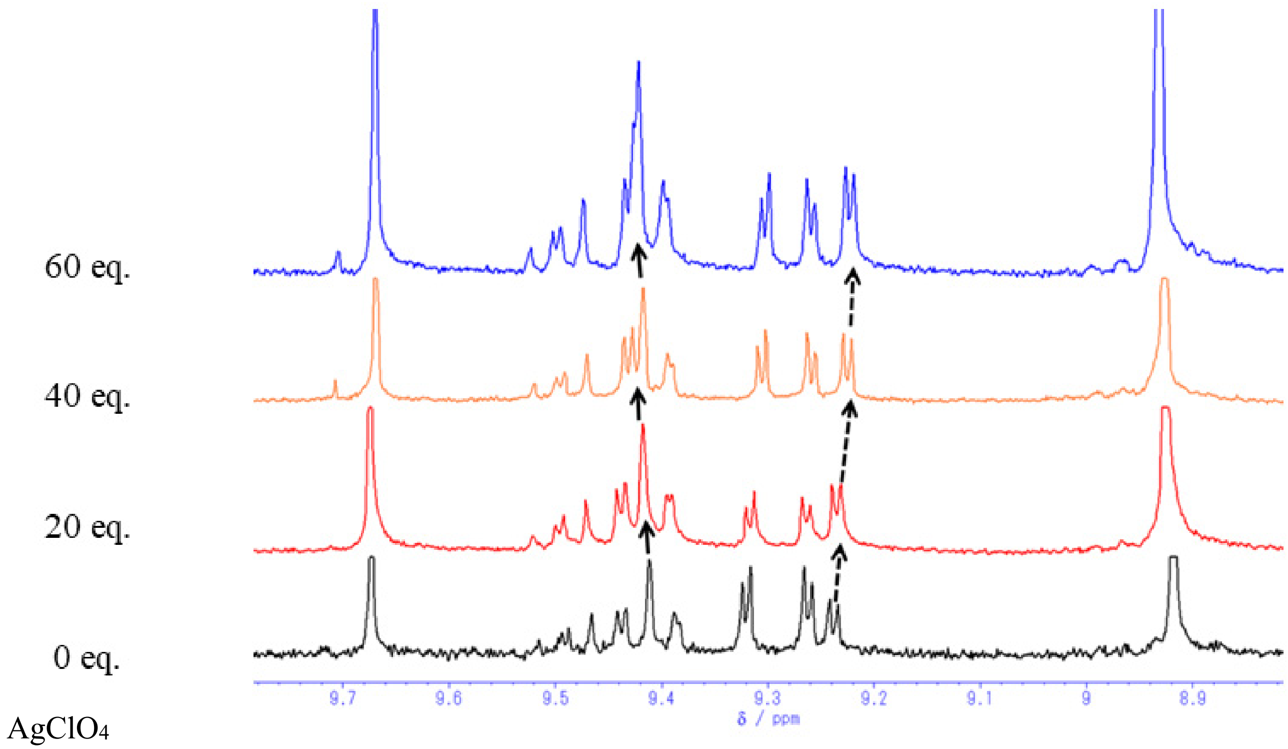

Figure S6 ^{1}H NMR spectra of compound **1** and **1**-$AgClO_4$ mixture (expanded). Solvent: THF-d_8.

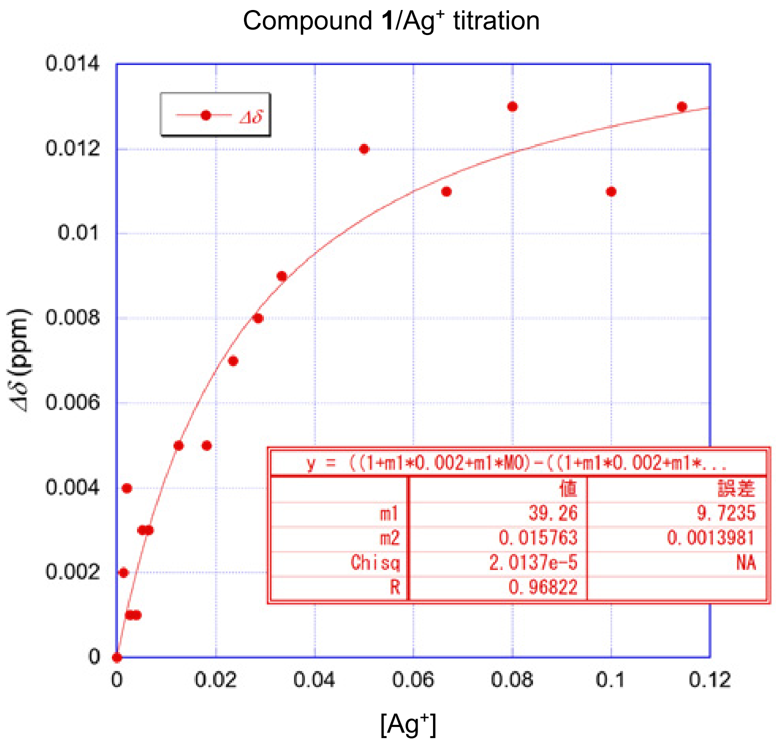

(a)

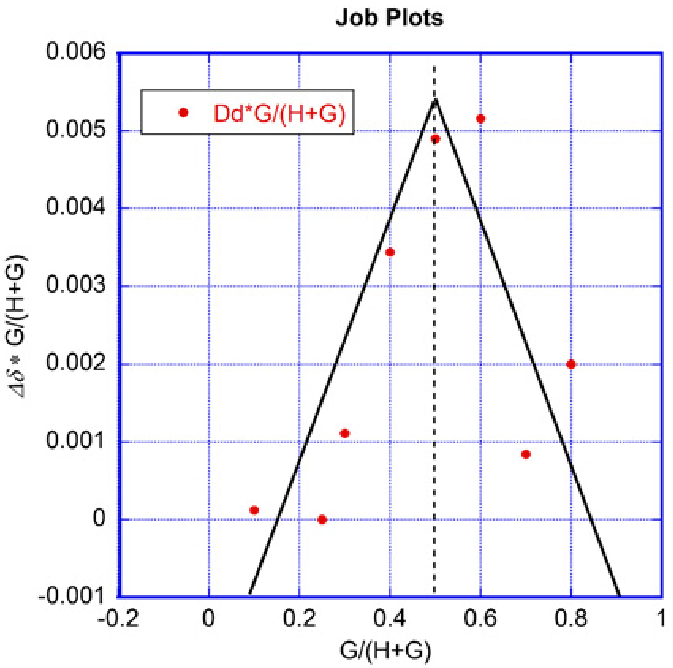

(b)

Figure S7 (a) Titration curve of compound **1** and $AgClO_4$ fitted using the nonlinear square method.

(b) Job plots.

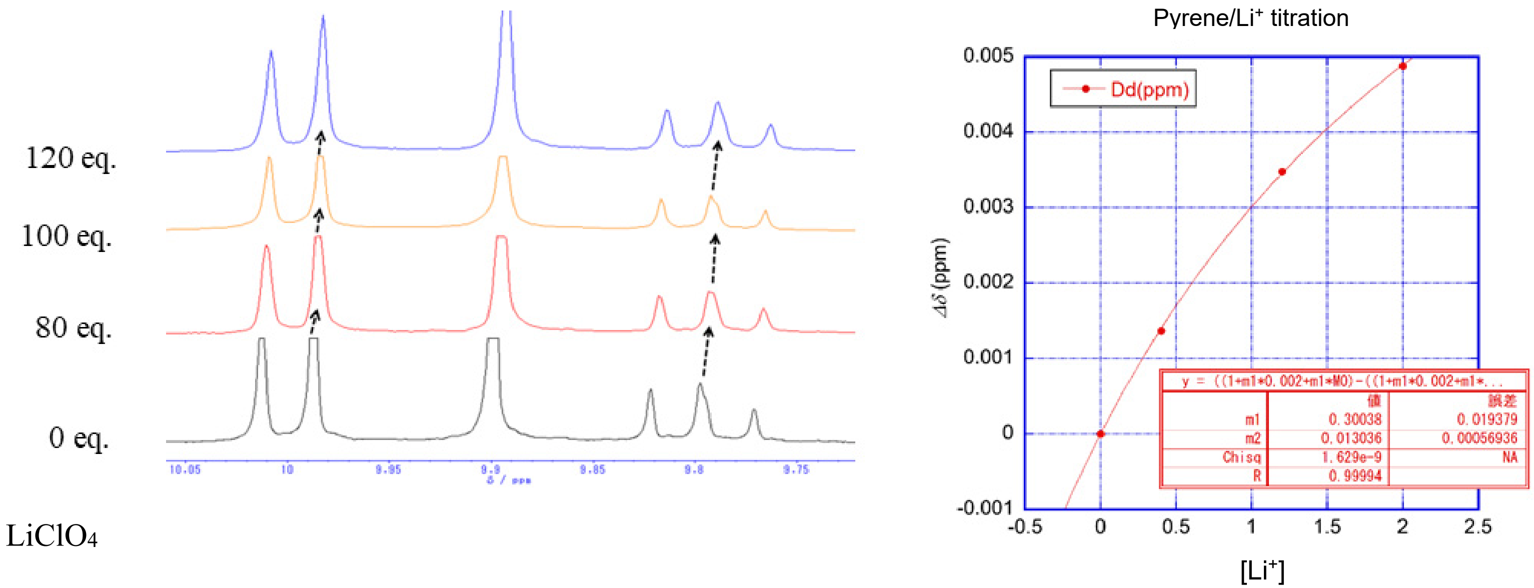

(a) (b)

Figure S8 (a) ^{1}H NMR spectra of pyrene and pyrene-$LiClO_4$ mixture (expanded). Solvent: THF-d_8.

(b) Titration curve.

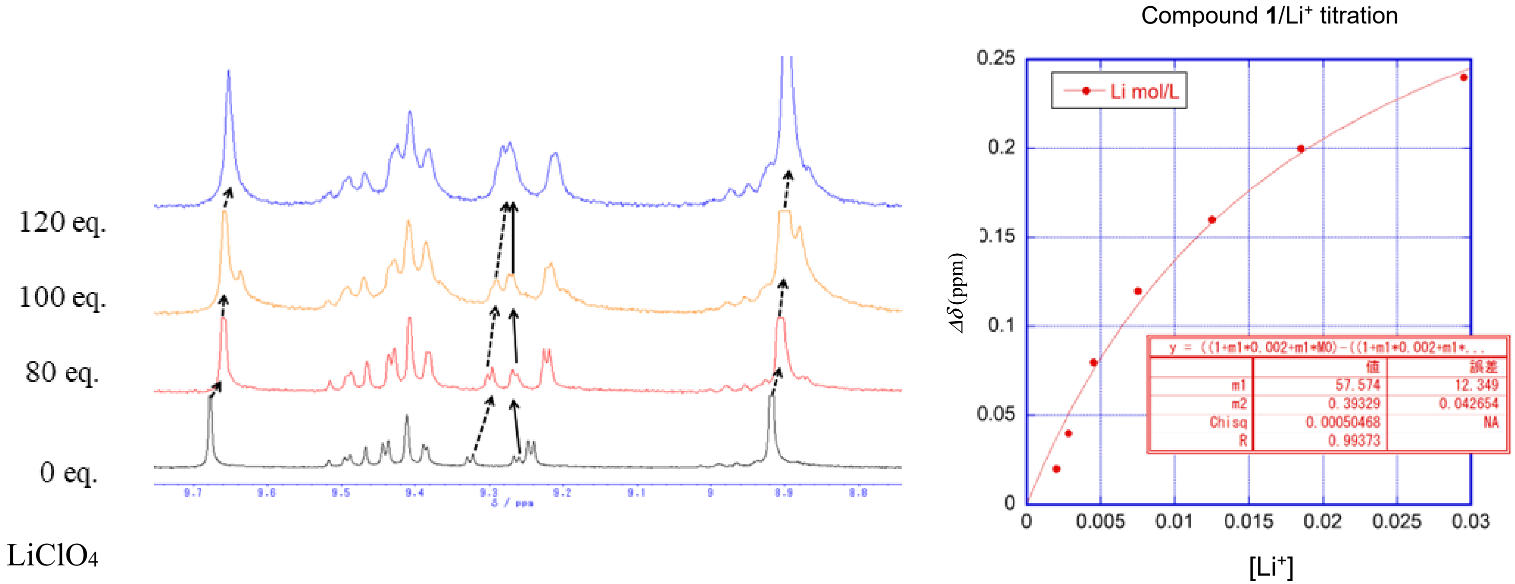

(a) (b)

Figure S9 (a) ^{1}H NMR spectra of compound **1** and **1**-$LiClO_4$ mixture (expanded). Solvent: THF-d_8.

(b) Titration curve.

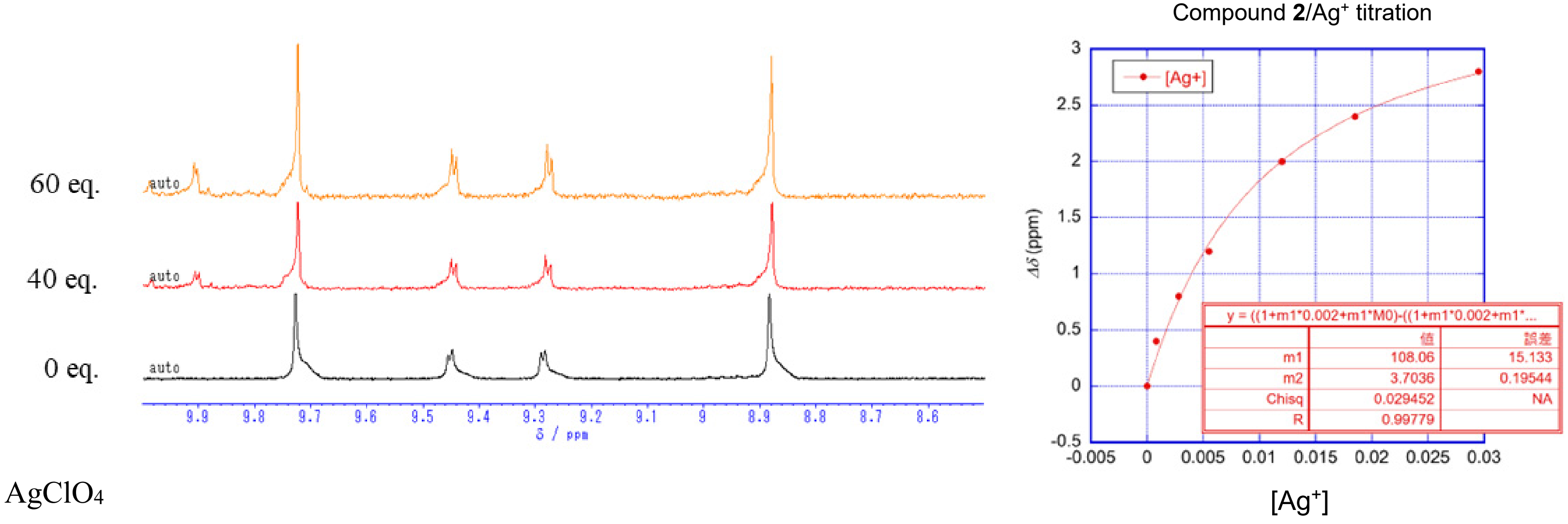

(a) (b)

Figure S10 (a) ^{1}H NMR spectra of compound **2** and **2**-$AgClO_4$ mixture (expanded). Solvent: THF-d_8

(b) Titration curve.

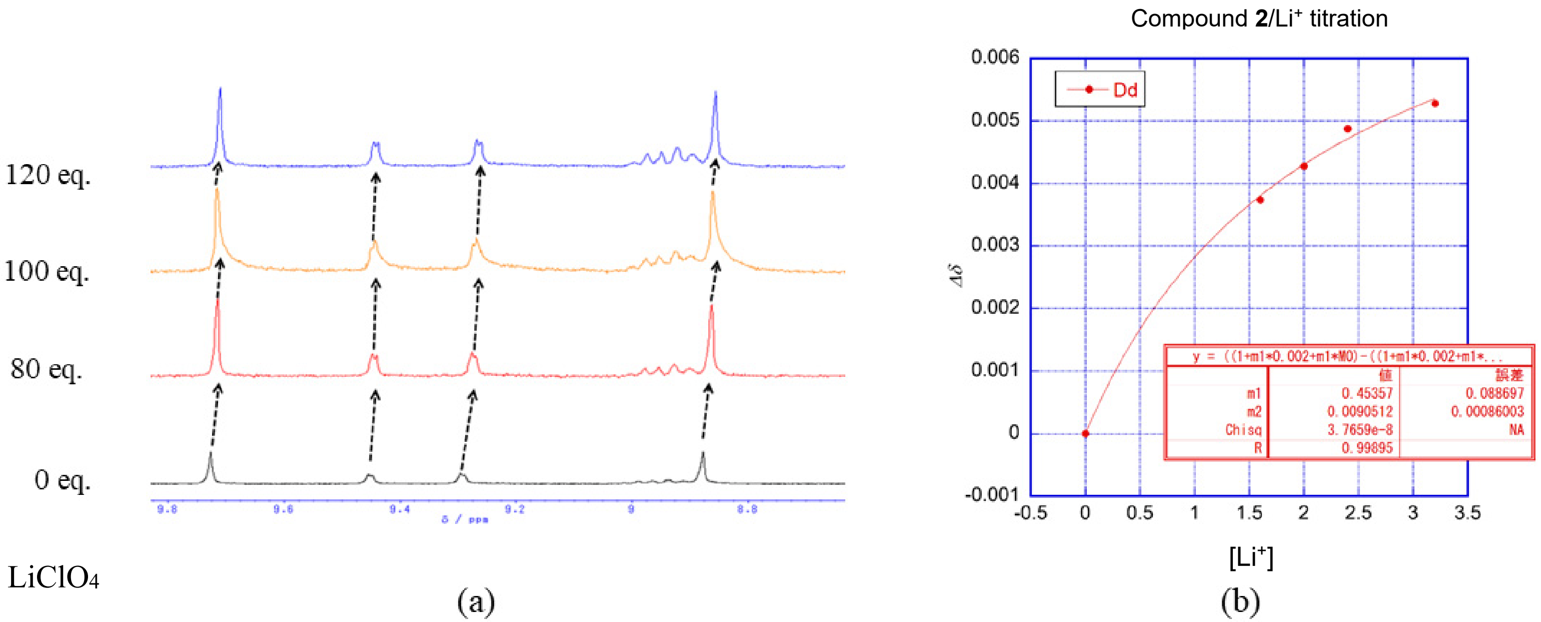

(a) (b)

Figure S11 (a) ^{1}H NMR spectra of compound **2** and **2**-$LiClO_4$ mixture (expanded). Solvent: THF-d_8.

(b) Titration curve.

4. DFT Calculations. Cartesian coordinates of 1 and 2.

DFT calculations for compound **1**.

B3LYP/6-31g(d,p)

# of imaginary frequencies = 0

Item	Value	Threshold	Converged?
Maximum Force	0.000010	0.000450	YES
RMS Force	0.000001	0.000300	YES
Maximum Displacement	0.000903	0.001800	YES
RMS Displacement	0.000222	0.001200	YES

Predicted change in Energy=-2.286834D-09

Optimization completed.

Stoichiometry C45H26O

Framework group C1[X(C45H26O)]

Deg. of freedom 210

Full point group C1 NOp 1

Input orientation:

-- Stationary point found.

Center Number	Atomic Number	Atomic Type	Coordinates (Angstroms) X	Y	Z
1	6	0	3.300797	-3.256257	2.113136
2	6	0	4.683348	-3.382863	2.231701
3	6	0	5.511953	-2.517884	1.523878
4	6	0	4.973261	-1.532202	0.692646
5	6	0	3.582825	-1.407890	0.602686
6	6	0	2.715174	-2.267985	1.307713
7	6	0	5.860630	-0.642163	-0.144393
8	6	0	5.143462	0.606533	-0.598546
9	6	0	3.745337	0.620980	-0.654845
10	8	0	2.998836	-0.454630	-0.211441
11	6	0	5.848508	1.741276	-1.007562

12	6	0	5.177486	2.862054	-1.487209
13	6	0	3.786719	2.845506	-1.565585
14	6	0	3.035477	1.734091	-1.153797
15	6	0	-5.120500	-0.879318	1.528793
16	6	0	-3.716716	-0.879553	1.512007
17	6	0	-3.028399	-2.110066	1.284712
18	6	0	-3.768329	-3.314464	1.078913
19	6	0	-5.171301	-3.264050	1.106237
20	6	0	-5.836151	-2.059061	1.328719
21	6	0	-1.603396	-2.135509	1.262709
22	6	0	-0.910943	-3.361891	1.037419
23	6	0	-1.681737	-4.558047	0.828897
24	6	0	-3.043159	-4.534953	0.849756
25	6	0	-2.940970	0.314477	1.713278
26	6	0	-1.580009	0.288900	1.690976
27	6	0	-0.858618	-0.934740	1.464048
28	6	0	0.542358	-0.982309	1.442042
29	6	0	1.229576	-2.186204	1.235056
30	6	0	0.490871	-3.360879	1.030758
31	6	0	-4.728948	3.387466	-1.634951
32	6	0	-3.342353	3.299603	-1.431496
33	6	0	-2.656712	2.114607	-1.839056
34	6	0	-3.382389	1.040865	-2.439001
35	6	0	-4.767904	1.175117	-2.620841
36	6	0	-5.430120	2.335952	-2.223431
37	6	0	-1.249380	2.002104	-1.644704
38	6	0	-0.559836	0.818069	-2.043682
39	6	0	-1.317935	-0.246911	-2.643873
40	6	0	-2.661877	-0.140005	-2.832189
41	6	0	-2.581553	4.358549	-0.824632
42	6	0	-1.236951	4.249432	-0.638237
43	6	0	-0.519753	3.070159	-1.043368
44	6	0	0.865147	2.936776	-0.870108
45	6	0	1.552203	1.781024	-1.271265
46	6	0	0.825122	0.729516	-1.847627
47	1	0	2.646398	-3.914718	2.676562

48	1	0	5.108142	-4.146559	2.876667
49	1	0	6.593315	-2.605614	1.602569
50	1	0	6.764994	-0.375916	0.417321
51	1	0	6.212919	-1.201616	-1.026520
52	1	0	6.934986	1.731887	-0.956359
53	1	0	5.732030	3.737156	-1.813238
54	1	0	3.258047	3.702615	-1.971845
55	1	0	-5.647428	0.056581	1.697589
56	1	0	-5.737663	-4.179356	0.951070
57	1	0	-6.922659	-2.038961	1.345829
58	1	0	-1.150090	-5.490707	0.654946
59	1	0	-3.610391	-5.449465	0.692455
60	1	0	-3.469105	1.250603	1.876290
61	1	0	-1.010687	1.203506	1.834015
62	1	0	1.101315	-0.065814	1.599638
63	1	0	1.016573	-4.295318	0.850689
64	1	0	-5.253721	4.289056	-1.327741
65	1	0	-5.323762	0.358698	-3.075360
66	1	0	-6.503121	2.421528	-2.373245
67	1	0	-0.790677	-1.149713	-2.941555
68	1	0	-3.219685	-0.957096	-3.283162
69	1	0	-3.107411	5.258887	-0.515312
70	1	0	-0.677824	5.061208	-0.178715
71	1	0	1.416447	3.748976	-0.402940
72	1	0	1.342901	-0.172075	-2.157892

Rotational constants (GHZ): 0.1057207 0.0645932 0.0440615

DFT calculations for compound **2**.

B3LYP/6-31g(d,p)

# of imaginary frequencies = 0

Item	Value	Threshold	Converged?
Maximum Force	0.000005	0.000450	YES
RMS Force	0.000001	0.000300	YES
Maximum Displacement	0.000441	0.001800	YES
RMS Displacement	0.000104	0.001200	YES

Predicted change in Energy=-5.124420D-09

Optimization completed.

Stoichiometry C58H32O2

Framework group C1[X(C58H32O2)]

Deg. of freedom 270

Full point group C1 NOp 1

Input orientation:

Center Number	Atomic Number	Atomic Type	Coordinates (Angstroms) X	Y	Z
1	6	0	5.667555	3.560359	-0.426800
2	6	0	7.054452	3.645996	-0.425670
3	6	0	7.805733	2.490638	-0.291180
4	6	0	7.193529	1.246732	-0.151950
5	6	0	5.803731	1.183878	-0.154269
6	6	0	5.013496	2.336799	-0.295902
7	6	0	8.025862	0.000485	0.003619
8	6	0	7.193530	-1.246347	0.154442
9	6	0	5.803724	-1.183535	0.156754
10	8	0	5.126415	0.000372	0.002765
11	6	0	7.805755	-2.490624	0.290177
12	6	0	7.054493	-3.646336	0.421708
13	6	0	5.667603	-3.560700	0.423286
14	6	0	5.013499	-2.336829	0.295474

15	6	0	-2.818311	1.434383	-1.132807
16	6	0	-1.424738	1.438526	-1.166231
17	6	0	-0.712117	2.333967	-0.328943
18	6	0	-1.423853	3.213707	0.523624
19	6	0	-2.819314	3.180595	0.520474
20	6	0	-3.526978	2.297769	-0.295964
21	6	0	0.712126	2.333966	-0.328944
22	6	0	1.423864	3.213708	0.523620
23	6	0	0.676149	4.103152	1.372848
24	6	0	-0.676136	4.103151	1.372850
25	6	0	-0.675652	0.550765	-2.017308
26	6	0	0.675656	0.550763	-2.017308
27	6	0	1.424745	1.438523	-1.166232
28	6	0	2.818318	1.434379	-1.132808
29	6	0	3.526986	2.297767	-0.295970
30	6	0	2.819325	3.180596	0.520466
31	6	0	-2.818318	-1.435771	1.133880
32	6	0	-1.424746	-1.439971	1.167298
33	6	0	-0.712124	-2.334193	0.328716
34	6	0	-1.423864	-3.212587	-0.525234
35	6	0	-2.819320	-3.179409	-0.522108
36	6	0	-3.527003	-2.297844	0.295691
37	6	0	0.712115	-2.334194	0.328715
38	6	0	1.423854	-3.212586	-0.525238
39	6	0	0.676136	-4.100711	-1.375850
40	6	0	-0.676149	-4.100712	-1.375848
41	6	0	-0.675657	-0.553398	2.019612
42	6	0	0.675652	-0.553400	2.019612
43	6	0	1.424739	-1.439974	1.167298
44	6	0	2.818312	-1.435775	1.133878
45	6	0	3.526995	-2.297846	0.295685
46	6	0	2.819310	-3.179407	-0.522116
47	6	0	-5.667545	3.560370	-0.426746
48	6	0	-7.054440	3.646011	-0.425607
49	6	0	-7.805725	2.490650	-0.291159
50	6	0	-7.193525	1.246738	-0.151980

51	6	0	-5.803727	1.183880	-0.154301
52	6	0	-5.013487	2.336803	-0.295892
53	6	0	-8.025862	0.000485	0.003517
54	6	0	-7.193535	-1.246341	0.154411
55	6	0	-5.803729	-1.183533	0.156723
56	8	0	-5.126415	0.000365	0.002689
57	6	0	-7.805763	-2.490611	0.290199
58	6	0	-7.054504	-3.646321	0.421771
59	6	0	-5.667613	-3.560689	0.423340
60	6	0	-5.013507	-2.336825	0.295483
61	1	0	5.067304	4.456028	-0.550220
62	1	0	7.542269	4.608753	-0.536785
63	1	0	8.891569	2.540889	-0.292032
64	1	0	8.692389	-0.105436	-0.862397
65	1	0	8.687258	0.106887	0.873551
66	1	0	8.891591	-2.540862	0.290859
67	1	0	7.542324	-4.609382	0.530231
68	1	0	5.067389	-4.456691	0.544520
69	1	0	-3.358870	0.740473	-1.767209
70	1	0	-3.364715	3.845909	1.184051
71	1	0	1.227166	4.778204	2.021813
72	1	0	-1.227152	4.778203	2.021818
73	1	0	-1.226915	-0.137241	-2.651479
74	1	0	1.226917	-0.137244	-2.651479
75	1	0	3.358876	0.740466	-1.767209
76	1	0	3.364729	3.845912	1.184039
77	1	0	-3.358868	-0.742797	1.769306
78	1	0	-3.364702	-3.843645	-1.186779
79	1	0	1.227151	-4.774748	-2.025872
80	1	0	-1.227165	-4.774749	-2.025868
81	1	0	-1.226922	0.133684	2.654782
82	1	0	1.226919	0.133681	2.654782
83	1	0	3.358862	-0.742804	1.769306
84	1	0	3.364689	-3.843641	-1.186791
85	1	0	-5.067292	4.456042	-0.550137
86	1	0	-7.542255	4.608774	-0.536685

87	1	0	-8.891561	2.540904	-0.292007
88	1	0	-8.687337	0.106881	0.873389
89	1	0	-8.692311	-0.105442	-0.862560
90	1	0	-8.891599	-2.540847	0.290884
91	1	0	-7.542338	-4.609361	0.530332
92	1	0	-5.067401	-4.456678	0.544603

Rotational constants (GHZ): 0.0943572 0.0284304 0.0229279
